# Body mass index and migraine: a survey of the Chinese adult population

**DOI:** 10.1007/s10194-012-0470-5

**Published:** 2012-07-19

**Authors:** Shengyuan Yu, Ruozhuo Liu, Xiaosu Yang, Gang Zhao, Xiangyang Qiao, Jiachun Feng, Yannan Fang, Xiutang Cao, Mianwang He, Timothy J. Steiner

**Affiliations:** 1 Department of Neurology, Chinese PLA General Hospital, Fuxing Road 28, Haidian District, 100853 Beijing, China; 2 Department of Neurology, Xiangya Hospital, Central-South University, Changsha, Hunan Province China; 3 Department of Neurology, The Fourth Military Medical University, Xian, Shaanxi Province China; 4 Department of Neurology, Affiliated Huashan Hospital of Fudan University, Shanghai, China; 5 Department of Neurology, The First Hospital of Jilin University, Changchun, Jilin Province China; 6 Department of Neurology, The First Affiliated Hospital of Sun Yat-sen University, Guangzhou, Guangdong Province China; 7 Department of Health and Economics, Chinese PLA General Hospital, Beijing, China; 8 Department of Neuroscience, Norwegian University of Science and Technology, Trondheim, Norway

**Keywords:** Headache, Migraine, Prevalence, Body mass index, Obesity

## Abstract

The objective of this study is to evaluate the association between body mass index (BMI) and migraine in general population Chinese cohort. This was a cross-sectional secondary analysis from a general population Chinese cohort of men and women of reproductive and post-reproductive age ranging between 18 and 65 years. Migraine was defined utilizing ICHD criteria. Body mass indices were calculated using measured height and weight and categorized based on the World Health Organizations criteria. The 1-year period prevalence of migraine was 9.3 %. No association was identified between migraine and those with a BMI < 30.0. Compared to those with normal BMI (18.5–23.0), those with BMI ≥ 30 (morbid obesity) had a greater prevalence of migraine (8.6 vs. 13.8 %, *p* = 0.000). Multivariate-adjusted odds ratio demonstrated that those with morbid obesity had a greater than twofold increased odds of migraine [OR 2.10 (1.39–3.12)] as compared to those with a BMI between 18.5 and 23.0. No association was found between obesity and migraine severity, frequency, or disability. Morbid obesity was associated with twofold increased odds of migraine in this Chinese men and women cohort of predominantly reproductive age.

## Introduction

Obesity is a major medical and public-health problem worldwide, affecting virtually all age and socioeconomic groups. Its prevalence is constantly increasing throughout the world [1, 2], and it threatens to overwhelm both developed and developing countries. In China, obesity, defined as body mass index (BMI) ≥23.0 kg/m^2^, affects almost half (44.6 %) of the adult population among adults age 40 years and above (men: 46.4 %; women: 43.6 %) [3].

Migraine is also a major public-health problem worldwide, although less well recognized. In China, its prevalence in adults is 9.3 % (men: 5.9 %; women: 12.8 %) [4], and it causes substantial illness and disability. Apart from their immediate impacts on well-being and ability to function, migraine and obesity are independent risk factors for cardiovascular disease [5, 6], comorbid with pain-related and psychiatric conditions [7–9] and lead to quality-of-life impairments [10, 11]. These associations add to their importance as causes of public ill-health. There are, in addition, suggestions that obesity and migraine are themselves associated. Several (predominantly Caucasian) general population studies have shown an association between migraine and obesity in those of reproductive age [12–15]. There were reports that obesity is associated with higher frequency and severity of headache attacks among individuals who have migraine [16–18].

We investigated these associations in a recently completed large nationwide cross-sectional survey of the Chinese population [4, 19], with the support of *Lifting The Burden*: the Global Campaign against Headache [20]. This was the first study evaluating the association between migraine and obesity in an Asian population. Through the comparison between Asia-Pacific population and Caucasian population, it helps for us to more thoroughly understand the association between obesity and migraine.

## Methods

### Data source

This was a secondary analysis of previous epidemiological survey data of primary headache disorders in mainland of China [4].

### Ethics

The study protocol was approved by the Chinese Ministry of Health and the ethics committee of the Chinese PLA General Hospital, Beijing, China.

### Previous epidemiological study

In 2009, we performed a door-to-door survey of 5,041 respondents throughout China to establish the prevalence of headache disorders [4, 19]. We included basic demographic information (habitation, age, gender, marital status, occupation and educational level). We employed a structured questionnaire, translated into Chinese from the English version developed by *Lifting The Burden* [20] for population-based studies and validated within the target population in a sub-study. We obtained a representative sample of the Chinese adult population using random-sampling software according to the EPI method established by WHO [21]. The method, described in detail elsewhere [4], is summarized as follows. China mainland has 27 provinces and 4 municipalities, which were merged into 24 units in a sampling frame based on the population size. From these 24 units, 35 cities or districts were randomly selected for sampling and, from each city or district, 20–30 towns or streets were randomly selected in proportion to the population. One village or community was randomly selected from each town or street, and one household was randomly selected from each village or community. The survey started from the selected household, and continued to include seven households along the same direction randomly decided by the interviewer. In each household, verbal consent was obtained first. Then the investigator listed all family members aged 18–65 years, and randomly selected one from the list to take part in the survey. The interviewers were neurologists employed by local hospitals, who lived close to the selected survey sites. They had completed a joint training programme on ICHD-II [22] provided by headache specialists and on survey methodology by epidemiology experts. Diagnoses were based on the ICHD-II criteria [22] and made algorithmically from questionnaire responses, a process that was validated in a subsample [4].

Among those with migraine, severity was assessed subjectively by respondents on a visual analogue scale (VAS) of 0–10, and “severe” headache was defined as VAS ≥8. “Frequent” headache was defined as occurring on 10–14 days/month (respondents with headache on ≥15 days/month were few and not included in this analysis). “Disabling” headache was defined as MIDAS grades II–IV [23].

### Anthropometric measurements

All body measurements used standard anthropometric protocols. Weight was recorded in kg using a calibrated digital scale. Height in meters was obtained from a fixed stadiometer. BMI was calculated as weight divided by square of height.

The classification of body weight followed the WHO guideline for the Asia-Pacific population [24]: underweight, BMI < 18.5; normal, BMI 18.5 to <23.0; overweight, BMI 23.0 to <25.0; obese, BMI 25.0 to <30.0; and morbidly obese, BMI ≥ 30.0 kg/m^2^.

### Statistical analysis

All demographic variables were categorized and presented as percentages. Age was also expressed as means ± standard deviations (SD). Migraine prevalence was estimated as percentages ± standard errors (SE). Independent Chi-squared tests were used to determine whether different weight categories had significant (*p* ≤ 0.05) differences in migraine prevalence, severity, frequency and/or disability. Multivariable-adjusted odds ratios (AOR) with 95 % confidence intervals (CI) were calculated by logistic regression taking into account of the following variables: age, gender, habitation, marital status, occupation, educational level.

## Results

Among the 5,041 survey participants, 12 with missing height or weight data were excluded, leaving 5,029 for the analysis [mean age: 43.6 ± 12.8 years; male 2,557 (mean age: 43.4 ± 12.9 years); female 2,472 (mean age 43.9 ± 12.8 years)]. Table 1 shows the distribution of demographic variables in the sample.

**Table 1:** Basic demographic data of the sample and migraine prevalence among different sub-groups

	*N*	%	Prevalence *n* (%)
Gender			
Male	2,557	50.8	152 (5.9 %)
Female	2,472	49.2	317 (12.8 %)
Habitation			
Urban	1,583	31.5	163 (10.3 %)
Rural	3,446	68.5	306 (8.9 %)
Marital status			
Unmarried	514	10.2	13 (2.5 %)
Married	4,342	86.3	438 (10.1 %)
Divorced	61	1.2	7 (11.5 %)
Widowed	112	2.2	11 (9.8 %)
Educational level			
Illiterate	406	8.1	47 (11.6 %)
Primary school	1,240	24.7	145 (11.7 %)
Secondary school	1,890	37.6	147 (7.8 %)
High school	953	19.0	82 (8.6 %)
College	540	10.7	48 (8.9 %)
Occupation			
Unemployed	428	8.5	40 (9.3 %)
Farmer	2,999	59.6	304 (10.1 %)
Worker	700	13.9	55 (7.9 %)
Student	129	2.6	4 (3.1 %)
Officer	654	13.0	61 (9.3 %)
Other	99	2.0	3 (3.0 %)
Soldier	20	0.4	2 (10.0 %)
Age (years)			
18–29	847	16.8	27 (3.2 %)
30–39	1,068	21.2	107 (10.0 %)
40–49	1,301	25.9	154 (11.8 %)
50–59	1,135	22.6	115 (10.1 %)
60–65	678	13.5	66 (9.7 %)

BMI categories are shown in Table 2, together with the estimated 1-year prevalence of migraine in each category. Overall, differences were significant (*χ* ^2^ = 14.661; *p* = 0.005). Virtually all of this difference was accounted for by the morbidly obese category, with a highly significantly increased prevalence of migraine (16.8 ± 2.7 %) compared with the normal weight category (8.6 ± 0.6 %; *χ* ^2^ = 14.427, *p* < 0.001; AOR = 2.095 [95 % CI 1.392–3.154], *p* < 0.001).

**Table 2:** 1 year migraine prevalence and adjusted odds ratios (AOR) for BMI categories

BMI	*N*	Prevalence *n* (%)	AOR (95 % CI)	*p*
Normal	2,469	212 (8.6 %)	Reference	
Underweight	262	27 (10.3 %)	1.22 (0.80–1.87)	0.359
Overweight	1,064	100 (9.4 %)	1.08 (0.84–1.39)	0.541
Obese	1,044	96 (9.2 %)	1.04 (0.80–1.34)	0.795
Morbidly obese	190	32 (16.8 %)	2.10 (1.39–3.15)	0.000
Total	5,029	467 (9.3 %)		

Overall *χ* ^2^ = 14.661

*p* = 0.005

Severity, frequency and disability levels among different BMI categories showed no significant differences (Tables 3, 4, 5). It’s worth noting that participants in the underweight category tended more frequently to have severe migraine (47.8 ± 10.4 vs. 27.9 ± 3.7 %; AOR = 2.32 [0.94–5.75]; *p* = 0.070) and were more likely to be disabled (70.8 ± 9.3 % vs. 50.0 ± 3.8 %; AOR = 2.34 [0.92–5.97]; *p* = 0.075) than those of normal weight.

**Table 3:** Proportions with, and adjusted odds ratios (AOR) for, severe migraine (VAS ≥8) among different BMI categories

BMI	Severe migraine *n*/denominator (%)	AOR (95 % CI)	*p*
Normal	41/147 (27.9 %)	Reference	
Underweight	11/23 (47.8 %)	2.32 (0.94–5.75)	0.070
Overweight	20/82 (24.4 %)	0.83 (0.44–1.57)	0.569
Obese	16/73 (21.9 %)	0.72 (0.36–1.43)	0.348
Morbidly obese	8/25 (32.0 %)	1.13 (0.45–2.86)	0.793
Total	96/350 (27.4 %)		

Overall *χ* ^2^ = 6.580

*p* = 0.160

**Table 4:** Proportions with, and adjusted odds ratios (AOR) for, frequent migraine attacks (10–14 days/month) among different BMI categories

BMI	Frequent migraine *n*/denominator (%)	AOR (95 % CI)	*p*
Normal	10/121 (8.3 %)	Reference	
Underweight	0/13 (0.0 %)	0.000	0.999
Overweight	5/65 (7.7 %)	1.03 (0.32–3.29)	0.960
Obese	8/53 (15.1 %)	2.11 (0.74–5.98)	0.161
Morbidly obese	1/18 (5.6 %)	0.64 (0.08–5.41)	0.680
Total	24/270 (8.9 %)		

Overall *χ* ^2^ = 4.208

*p* = 0.379

**Table 5:** Proportions with, and adjusted odds ratios (AOR) for, disabling migraine (MIDAS grades II–IV) among different BMI categories

BMI	Disabling migraine *n*/denominator (%)	AOR (95 % CI)	*p*
Normal	87/174 (50.0 %)	Reference	
Underweight	17/24 (70.8 %)	2.34 (0.92–5.97)	0.075
Overweight	46/89 (51.7 %)	1.09 (0.65–1.82)	0.751
Obese	41/84 (48.8 %)	0.98 (0.57–1.67)	0.940
Morbidly obese	14/27 (51.9 %)	1.16 (0.51–2.65)	0.718
Total	205/398 (51.5 %)		

Overall *χ* ^2^ = 3.994;

*p* = 0.407

## Discussion

First we emphasize the need, when analyzing and interpreting these data, to take into consideration the WHO body weight classification guideline specifically for the Asia-Pacific population [24]. Doing this, we found a link between obesity and migraine prevalence rather than severity, frequency or disability. Morbidly obese respondents were twice as likely as those of normal weight to have migraine, the difference being highly significant. However, other BMI categories (underweight, overweight and obese) showed little relationship with prevalence. There were matching trends in the underweight to have migraine of greater severity and be more disabled.

The relationship between migraine and obesity was first evaluated in a clinic-based study, which found obese patients were three times as likely as age-matched normal-weight controls to have migraine [25]. Ford et al. [12] found that those who were underweight (BMI < 18.5) or obese (BMI ≥ 30) were at higher risk for having severe headaches or migraine compared with those of normal weight. Peterlin et al. [13] found, in men and women aged 20–55 years, that higher migraine prevalence was associated with both total and abdominal obesity. And, this was the first study which suggested and clearly demonstrated that older individuals or those of post-reproductive age who have migraine do not have an association with obesity while those of reproductive age do, which also suggested that both obesity and migraine are modulated by reproductive status [26, 27]. The finding that migraine and obesity are associated in those of reproductive age by Peterlin et al. [13] was also later supported by data from Vo et al. [15] and Robberstad et al. [14]. Vo et al. [15] found a significant association between migraine and obesity and that the odds of migraine increased with increasing obesity status. Robberstad et al. [14] found that recurrent headache was associated with overweight (odds ratio [OR] = 1.4, 95 % CI 1.2–1.6, *p* < 0.0001).

The role of race played in the association between migraine and obesity is not clear at present. In contrast to the Peterlin et al. [13] study and Vo et al. [15] study which were predominantly Caucasian population, our study has shown that there is an association between morbid obesity and migraine in a Asian population for the first time.

The reproductive status influence should be taken into consideration. While two studies done by Winter et al. [28] and Mattsson [29] evaluating populations of only post-reproductive age showed no association between migraine and obesity. Comparing with Peterlin et al. [13] study, Mattsson [29] study mainly focus on post-menopausal age group. Both reproductive age and post-menopausal age groups were included in our study, which might dilute our findings.

Bigal et al. [16, 18] found that obesity, while not associated with increased prevalence of migraine, was related to frequent headache attacks (10–14 headache days/month), headache disability and likelihood of experiencing severe headache. Queiroz et al. [30] found no significant relationship between migraine and BMI. Keith et al. [31] found that obese women had increased risk of headache, but not specifically migraine. Téllez-Zenteno et al. [32] found that there was no association between the disability and severity of migraine and BMI.

In conclusion, several general population group studies have shown a clear association between obesity and migraine in those of reproductive age group [13–15]. In our study, we found migraine prevalence was significantly raised in the morbidly obese group, and this was a substantial and statistically significant increase. We also observed that there was a weak link between being underweight and migraine severity and disability.

Our study has several strengths. It was the first China nationwide study on headache of a population-based randomly selected sample, and was also the first migraine and obesity study in Asians, utilizing properly utilized measured BMIs. The sample was large (>5,000 respondents). Migraine was diagnosed by physician interviewers using a questionnaire based on the ICHD-II criteria. Anthropometric measurements were performed by the same interviewers, guaranteeing the accuracy of body height and weight information. While information on migraine severity and frequency depended, unavoidably, on subjective self-reporting by respondents, disability was estimated through the widely used MIDAS score [23]. Furthermore, information about a large number of potential confounders was available, allowing us to make due adjustments in detecting any association between BMI and migraine.

Some limitations should also be considered. Despite our ability to control for a large number of potential confounders, we could not know of other comorbidities, including psychological, that might also be confounders, and we cannot exclude the possible influence of these since our study was observational. This study was also limited by the inclusion of post-reproductive aged individuals in light of the studies by Peterlin et al. [13], Vo et al. [15] and Robberstad et al. [14]. In addition, our study was cross-sectional, not allowing us to determine any time sequence in the association between BMI and migraine. Otherwise, it is worth noting that the observation can be biased just because of the reduced sample size in the group of morbid obesity, and this should be taken into consideration while analyzing the results.

Although at present, there can be no definitive conclusion about the relationship between migraine and obesity, it is almost certain that the two are associated, in a probably complex way. The reasons for inconsistency in findings are not entirely clear. While we found, in the Chinese population, that morbid but not lesser degrees of obesity is associated with increased migraine prevalence but not severity, frequency or disability, studies of other populations have found evidence that obesity is associated with more frequent and severe migraine [16–18] rather than an increased risk of having migraine. It may be that there are differences based on the culture or ethnicity. Certainly, any underlying mechanism linking migraine and obesity is not evident. Further population studies are needed to sort this out; whether or not a link exists is an important question both for its public-health implications and for better understanding the pathophysiology of these disorders.
